# Association Between Telomere Shortening and Erythropoietin Resistance in Patients with Chronic Kidney Disease Undergoing Hemodialysis

**DOI:** 10.3390/ijms26073405

**Published:** 2025-04-05

**Authors:** Blanca Olivia Murillo-Ortiz, Marcos Javier Romero-Vázquez, Angélica Jeanette Luevanos-Aguilera, Paulina Monserrat Meza-Herrán, Edna Montserrat Ramos-Rodriguez, Sandra Martínez-Garza, Mario Murguia-Perez

**Affiliations:** 1 Clinical Epidemiology, Research Unit, OOAD Guanajuato, Mexican Institute of Social Security, León 37328, Guanajuato, Mexico; marcsjav@hotmail.com (M.J.R.-V.); aj.luevanosaguilera@gmail.com (A.J.L.-A.); dra.pmezher@gmail.com (P.M.M.-H.); samartin30@yahoo.com (S.M.-G.); 2 Department of Hemodialysis, Hospital General Regional No. 58, Institute Mexican of Social Security, León 37268, Guanajuato, Mexico; ednaramro@hotmail.com; 3 Departamento de Anatomía Patológica, UMAE Hospital de Especialidades No. 1, Centro Médico Nacional Bajío, Instituto Mexicano del Seguro Social, León 37328, Guanajuato, Mexico; 4 Laboratorio de Anatomía Patológica e Inmunohistoquímica Especializada DIME, Hospital Médica Campestre, León 37160, Guanajuato, Mexico

**Keywords:** telomere shortening, erythropoietin resistance, anemia, hemodialysis

## Abstract

The relationship between telomere shortening and patients with chronic kidney disease (CKD) has recently been investigated. Although most patients respond adequately to erythropoiesis-stimulating agents (ESAs), approximately 10% do not, and this is referred to as ESA resistance. The aim of our study was to investigate the relationship between telomere shortening and erythropoietin resistance in patients with CKD on hemodialysis. This cross-sectional, comparative, analytical, and observational study was conducted in patients of both sexes over 18 years of age diagnosed with CKD. Two groups of patients were identified. The first group consisted of 40 patients receiving erythropoiesis-stimulating agents with erythropoietin resistance. The second group consisted of 40 patients with the same characteristics but without erythropoietin resistance. Telomere length was measured by real-time PCR. Eighty patients were included in the study. Mean hemoglobin levels were lower in the erythropoietin resistance group (8.8 ± 1.67 vs. 11.95 ± 1.81, *p* = 0.001). Differences were observed in hematocrit and albumin levels, which were lower in patients with erythropoietin resistance, while PTH levels were higher in this group (788 ± 538.47 vs. 535.65 ± 603.06, *p* = 0.001). A significant difference in telomere length (T/S) was observed between the two groups, with shorter telomere length in the erythropoietin resistance group (0.45 ± 0.04 vs. 0.56 ± 0.03, *p* = 0.01). Telomere shortening may be associated with anemia and erythropoietin resistance in patients with CKD undergoing hemodialysis. This relationship suggests the need to explore whether telomere length recovery improves the response to ESAs.

## 1. Introduction

Anemia in patients with CKD is of multifactorial origin; however, the most studied cause is the relative deficiency in erythropoietin (EPO) secretion. Erythropoietin production occurs in the peritubular capillaries of the endothelial cells in the kidneys, under a feedback mechanism triggered by decreased blood oxygen levels. EPO binds to erythroid progenitor cell receptors in the bone marrow. The absence of erythropoietin results in programmed cell death, or apoptosis [1].

The indication for treatment with erythropoiesis-stimulating agents is when hemoglobin is between 9 and 10 g/dL, with an initial dose of alpha or beta erythropoietin being 20 to 50 U/kg of body weight three times a week. Hemoglobin levels should be monitored every four weeks, with the goal of increasing by 1 to 2 g per deciliter per month. If there is no increase and levels remain below 11 g/dL, the total dose should be increased by 25%. The recommended goal is to maintain hemoglobin levels between 11 and 12 g/dL or hematocrit between 33 and 36% [2].

Although most patients respond adequately to erythropoiesis-stimulating agents, approximately 10% do not, and this is referred to as ESA resistance [3]. According to the KDIGO 2012 guidelines, ESA resistance was defined if at least one of the following occurred: two dose increases of up to 50% above the previously stable dose were required to maintain a stable Hb concentration, or a significant decrease in hemoglobin occurred despite a constant ESA dose. A failure to increase the Hb level to greater than 11 g/dL and a sudden rapid decrease in Hb level at the rate of 0.5 to 1.0 g/dL/wk. In addition, patients did not have nutritional deficiencies or hematologic or bleeding disorders. [2].

There are risk factors associated with ESA resistance, including absolute or functional iron deficiency [4]. In addition, inflammation is increased in patients with chronic kidney disease and low levels of serum albumin [5].

The activation of the immune system redirects iron transport from the site of erythropoiesis to the reticuloendothelial system [6], inhibits erythroid progenitor proliferation and differentiation, suppresses erythropoietin production, induces a deficient response to erythropoietin, and accelerates the destruction of erythrocytes bound to immune complexes or immunoglobulins [7]. Folate and vitamin B12 deficiency have been associated with resistance to erythropoiesis-stimulating agents [8].

Secondary hyperparathyroidism, with altered renal calcium and phosphorus homeostasis, affects hematopoietic cells in the bone marrow that express calcitriol receptors, inducing the proliferation and maturation of erythroid progenitor cells and affecting erythropoiesis [9]. Excess parathyroid hormone (PTH) can induce bone marrow fibrosis, reducing the space for erythropoiesis; this is due to excessive bone resorption and inhibition of osteoblastic activity, in addition to fibroblast proliferation caused by elevated PTH levels. This parathyroid hormone alteration has been associated with resistance to the effects of erythropoiesis-stimulating agents [10]. Secondary hyperparathyroidism is a less recognized, but potentially significant, cause of renal anemia in chronic kidney disease [11].

Trunzo JA and colleagues analyzed 37 patients with chronic kidney disease, erythropoietin resistance, and secondary hyperparathyroidism who underwent parathyroidectomy. They observed an increase in hemoglobin and hematocrit levels and a corresponding decrease in ESA dose. However, they concluded that parathyroidectomy should not be performed routinely but only in cases where there is no response to medical treatment [12].

A recently investigated aspect is the relationship between telomere shortening in patients with chronic kidney disease [13]. Telomeres are specialized nucleoproteins located at the linear ends of chromosomes. After each cell division, a small portion of the terminal chain is lost. When the telomere reaches a critical length, the cell is programmed to die, entering apoptosis, or a permanent cell cycle inhibition called cellular senescence, where they remain metabolically active but refractory to mitogenic stimuli. This state can be accelerated by exposure to oxidative stress, which induces telomere attrition with shortening of their sequence [14].

Telomeres are lengthened by the enzyme telomerase, which is active in immature, undifferentiated cells and some mature cells, such as lymphocytes and hematopoietic cells. In telomere disorders, mutations in the genes responsible for telomere maintenance and repair lead to organ dysfunction, including bone marrow failure and an increased risk of cancer. In the hematopoietic cells of patients with these alterations, telomere shortening causes a decrease in the number of hematopoietic cells, as well as a qualitative deficiency in the regeneration of these cells [15]. Samples with a T/S ratio greater than 1.0 have an average telomere length longer than that of the standard DNA, while samples with a T/S ratio less than 1.0 have a shorter average telomere length. There are reports on telomere length in healthy subjects Bhatt et al. reported a T/S of 0.94 ± 0.01 in healthy subjects aged 39.26 ± 12.48 years [16]. Nguyen et al., in a study conducted in a sample of healthy subjects including children and their parents, reported T/S values in fathers: 0.82 ± 0.36 at age 43.4 ± 5.3 years, in mothers 0.81 ± 0.38 at age 44.2 ± 5.7 years, and in children T/S: 1.09 ± 0.56 at age 12 ± 0.4 years [17].

In chronic kidney disease, there is an increase in the synthesis of proinflammatory cytokines and oxidative stress, which causes telomere/telomerase dysfunction [18,19]. In recent years, several studies have associated telomere length with chronic kidney disease [20]. Animal studies have demonstrated a relationship between telomere length in various organs—such as heart, kidney, and liver—and the progression of renal function impairment [21,22,23,24,25]. Additionally, there is evidence of telomere shortening in renal cells exposed to high glucose levels [26].

De Vusser et al. analyzed 40 kidney donors to assess intrarenal telomere length. Among these 40 subjects, high-quality DNA from leukocytes was available in 32 cases, while high-quality DNA from biopsies was obtained from all 40 kidneys. All 40 biopsies included in this cohort met the Banff 1997 criteria for histological evaluation.

Intrarenal telomere length, clinical demographics, and renal histology were measured. The mean T/S ratio for intrarenal telomere length was 0.07 ± 0.09 (range: −0.16–0.24). Intrarenal telomere length correlated significantly with leukocyte telomere length (r = 0.4, *p* = 0.001). Shorter intrarenal telomere length was associated with a history of hypertension (log T/S ratio: 0.04 ± 0.02 vs. 0.10 ± 0.02 in individuals with and without hypertension, respectively; *p* = 0.05), but not with other demographic factors. In this cohort, shorter intrarenal telomere length was significantly associated with the presence of arteriosclerosis (log T/S ratio: −0.04 ± 0.06 vs. 0.08 ± 0.01 in individuals with vs. without arteriosclerosis, *p* = 0.007) [27].

Aging is a natural process characterized by telomere shortening; however, some chronic diseases have shown signs of premature and accelerated aging. Telomere sequence loss has been associated with anemia in patients with chronic kidney diseases [28] and hematological diseases such as aplastic anemia, myelodysplastic syndromes, and Fanconi anemia [29,30]. This is explained by the fact that this shortening in the bone marrow impairs the function and proliferation capacity of hematopoietic cells, leading to anemia [31].

There is no evidence directly linking PTH levels to telomere dysfunction; however, secondary hyperparathyroidism has been reported to play a critical role in renal anemia through multiple pathways. The classic theory suggests that excessive secretion of PTH leads to bone marrow fibrosis, resulting in impaired erythropoiesis. This possibility was first proposed by Rao et al., who demonstrated that patients with a poor response to EPO exhibited a higher percentage of osteoclastic and eroded bone surfaces, as well as a greater degree of bone marrow fibrosis, in association with higher PTH levels compared to patients with a good response [32].

Analysis of telomere length shortening will help to know the possible effect on erythropoiesis, and the apparent association with PTH levels seems to be a factor related to bone marrow fibrosis. Gaweda et al. reported a modest but significant association between higher PTH levels and decreased erythropoietic response [33].

Treatment aimed at modulating telomerase by gene therapy must meet two polarizing requirements to achieve different therapeutic outcomes: anti-aging/regenerative applications and anticancer applications, which are achieved by suppressing multiple genes involved in telomere maintenance (e.g., telomerase, telomerase RNA components, and the sheltering complex) [34]. Upregulation and downregulation of telomerase-related genes have shown promising therapeutic results for anti-aging/regenerative and anticancer applications [35].

Experiments in cell and animal models provide proof of concept for the feasibility of telomerase activation approaches to counteract telomere shortening and its consequences. The successful use of telomerase gene therapy in animal models of aging and short telomere-related diseases paves the way for the development of therapeutic telomerase treatments in human aging and associated diseases [36].

To date, it has not been investigated whether erythropoietin-resistant CKD patients have telomere shortening to explore the possible relationship between telomere shortening and CKD progression. The study is based on a comparative approach between EPO-responsive and non-responsive CKD patients to determine whether significantly shorter telomeres and higher PTH levels in CKD patients are associated with EPO resistance. Investigating the possible causes of EPO resistance is important not only to elucidate the pathogenesis of CKD complications but also to provide a potential new therapeutic approach.

## 2. Results

Eighty patients were included in the study, of which thirty-eight were female (47.5%) and forty-two were male (52.5%). The mean age was 34 ± 14.7 years (case group) and 38 ± 11.6 years (control group), *p* = 0.873. Table 1. The causes of chronic kidney disease for both groups were chronic kidney disease of unknown etiology in 48 patients (60%), glomerulonephritis in 20 patients (25%), polycystic kidney disease (5%), bilateral renal hypoplasia (5%), and obstructive uropathy in 4 patients (5%). The mean duration of hemodialysis was 5 years (95% CI 4.64–7.01). All patients received anti-PTH therapy (calcium carbonate 1–1.5 g/day), calcitriol 0.25 mcg every 24 h, sevelamer 2400 mg/day. Recombinant human epoetin alfa, between 20 and 50 U/kg of body weight, was administered three times a week. The mean hemoglobin levels were lower in the group with erythropoietin resistance (8.8 ± 1.67 vs. 11.95 ± 1.81, *p* = 0.001). Statistically significant differences were observed in hematocrit and albumin levels, which were lower in patients with erythropoietin resistance, while PTH levels were higher in this group (788 ± 538.47 vs. 535.65 ± 603.06, *p* = 0.001). CRP did not differ between groups (3.6 ± 2.80 vs. 3.46 ± 2.34, *p* = 0.70).

The erythropoietin units administered per week were higher in the group with erythropoietin resistance (24,000 ± 4258 vs. 4000 ± 2783, *p* = 0.01). Table 2. A significant difference in telomere length (T/S) was observed between the two groups, with shorter telomere length in the group with erythropoietin resistance (0.45 ± 0.04 vs. 0.56 ± 0.03, *p* = 0.01). Multivariate logistic regression analysis showed that telomere shortening was independently associated with erythropoietin resistance (*p* = 0.001), albumin (*p* = 0.001), and hemoglobin (*p* = 0.005), while it was not significantly associated with CRP (*p* = 0.554) or PTH (*p* = 0.801).

## 3. Discussion

Recent studies have described various associated factors, such as the prospective, observational Dialysis Outcomes and Practice Patterns Study (DOPPS), a large group of patients with anemia that includes many international dialysis centers with a wide range of anemia treatment practices over the years. It analyzes hemoglobin, transferrin saturation, and serum ferritin, as well as ESA and intravenous iron doses [37,38]. In our study, we observed that patients with erythropoietin resistance had lower hemoglobin levels despite having elevated ferritin levels in both groups.

Karaboyas A et al. recently demonstrated in a prospective study that an acute increase in the inflammatory marker CRP is followed by a decrease in hemoglobin in response to ESAs [39]; in our study, the elevated CRP levels between the two groups of patients may be related to other described factors such as albumin levels and time on hemodialysis [40]. Ferritin was also not different, indicating that the inflammatory state is equally present in both groups.

Hyporesponsiveness to ESA has been associated with a higher risk of mortality, although it is not certain whether this is due to complications resulting from increased ESA doses in response to low hemoglobin concentrations, various unmeasured confounding factors, or a combination of these factors [41]. In the present study, we observed that patients with lower hemoglobin levels had lower albumin levels and higher PTH levels.

Secondary hyperparathyroidism is a common complication of chronic kidney disease due to decreased renal function, leading to increased serum phosphorus and low serum calcium levels, triggering increased secretion of PTH. This overproduction of PTH, along with vitamin D deficiency, causes a mineral bone disorder resulting in defective bone mineralization, vascular growth, soft tissue calcification, and bone marrow fibrosis [42]. The fibrosis production in the bone marrow caused by increased PTH adversely affects erythroid progenitor cells, thus exacerbating anemia in CKD patients [9]. Linking hematopoietic defects to fibrosis is crucial, involving other factors in the hematopoietic system that affect cell proliferation. We observed that patients with resistance to Erythropoiesis had higher PTH levels. Anemia might be a cardinal sign in individuals whose hematopoietic cells’ telomere length (HC TL) is too short to maintain daily erythropoiesis. This is shown by rare diseases known collectively as telomere biology disorders (TBDs) [43].

The most interesting finding of our study was the observation of a significant difference in the T/S ratio, demonstrating a shorter telomere length in CKD patients on hemodialysis who present resistance to erythropoietin.

We observed that patients undergoing renal replacement therapy with hemodialysis for longer durations are exposed to inflammation, uremia, and low albumin levels. Secondary hyperparathyroidism is considered an important factor contributing to the degree of bone marrow fibrosis, which impairs the hematopoietic response and requires higher doses of EPO to achieve an adequate hematopoietic effect [11]. Although the relationship between the T/S ratio and erythropoietin resistance has not been widely addressed in the literature, telomere shortening could lead to a reduced hematopoietic response, potentially increasing resistance to EPO.

Patients with telomere shortening may present with multisystem findings but often suffer from bone marrow failure (BMF), manifested by aplastic anemia and neutropenia. The development of aplastic anemia in TBDs provides evidence that critically short telomeres can curtail replication of human somatic cells in vivo [44].

Regarding the presence of anemia and biological age, it is known that approximately 10% of people over the age of 65 and more than 20% of those over the age of 85 have anemia. Interestingly, in one-third of these cases, the cause of anemia remains unexplained [45]. There is strong evidence for a correlation between telomere length and the number of circulating erythrocytes. These findings suggest that it is possible that the unexplained anemia in these elderly individuals is due in part to a decline in telomere-dependent erythropoiesis [46].

Recent research in experimental mouse models confirms that, importantly, all of these defects were reversed with the reactivation of telomerase activity, leading to the restoration of erythroblasts and erythroid progenitors in the bone marrow, as well as improved erythroid cell counts and hemoglobin levels in the peripheral blood. The researchers concluded that defective hematopoiesis, particularly erythropoiesis, resulting from telomerase loss can be reversed by restoring telomerase activity [47].

Androgen therapy has been used as a first-line treatment for aplastic anemia. A recent study in mice that developed full-blown aplastic anemia induced by short telomeres showed that androgen therapy rescued telomere attrition [48], suggesting that telomerase activation may indeed be a treatment option for diseases associated with defective telomere maintenance.

In addition, the use of telomerase activation has attracted the interest of commercial companies. For example, the low potency telomerase activator TA-65 (a bioactive compound isolated from the herb Astragalus membranaceus) has been shown to slightly increase telomere length in mice, zebra finches, and humans [34]. It is possible that anemia associated with aging may result in part from telomere shortening. Telomere length is extremely short in patients with erythropoietin resistance compared to those with longer telomeres who did not have erythropoietin resistance.

The observation that reactivation of telomerase not only reverses anemia but also restores homeostasis in other hematopoietic cell lineages suggests a potential therapeutic approach, for example, through TERT mRNA delivery [49].

Saraswati et al. contribute to a better understanding of the role of dysfunctional telomeres in the occurrence of profibrotic alterations leading to renal fibrosis. They observed down-regulation of the angiogenesis pathway with loss of CD31/PECAM+ and VEGFR2+ endothelial cells, as well as increased vascular permeability and hypoxia in kidneys lacking TRF1 in fibroblasts [50].

With increasing research into the associations between telomeres and CKD, new strategies for the management and treatment of CKD are on the horizon. The relationship between telomere length in leukocytes and renal cells could also serve as a diagnostic biomarker for the early detection of CKD.

This study has some limitations because it is a small, cross-sectional study; it is necessary to increase the number of patients to explore whether telomere length recovery improves the response to ESAs. As our analysis was based on univariate comparisons, it is possible that the observed associations are influenced by confounding variables. Multivariate analysis is required to assess whether telomere shortening is independently associated with ESA resistance.

In perspective, more research is needed to develop therapeutic treatments targeting telomerase in human aging and associated diseases. One area of investigation could be its effect on chronic kidney disease patients with erythropoietin resistance.

## 4. Materials and Methods

A cross-sectional, comparative, analytical, and observational study was conducted on patients of both sexes over 18 years of age diagnosed with chronic kidney disease and receiving erythropoiesis-stimulating agents, from the hemodialysis service at the Unidad Médica de Alta Especialidad No. 1 Bajío and Hospital General Regional No. 58 of the Instituto Mexicano del Seguro Social. Once the inclusion criteria were met and informed consent was obtained, two groups of patients were formed, both with chronic kidney disease on hemodialysis and receiving erythropoiesis-stimulating agents. The total population of patients screened to find the 40 patients with ESA resistance was n = 410, from the 5 shifts that exist per day in each hemodialysis service of the two treatment centers.

### 4.1. Inclusion Criteria

The study included patients aged ≥ 18 years with chronic kidney disease on hemodialysis. The first group (cases) consisted of 40 patients receiving erythropoiesis-stimulating agents with erythropoietin resistance as defined by the KDIGO 2012 guidelines, with an initial dose of alpha- or beta-erythropoietin between 20 and 50 U/kg body weight administered three times a week. ESA resistance was defined if at least one of the following occurred: two dose increases of up to 50% above the previously stable dose were required to maintain a stable Hb concentration, a significant decrease in hemoglobin occurred despite a constant ESA dose, or an inability to raise hemoglobin levels above 11 g/dL. In addition, patients did not have nutritional deficiencies or hematologic or bleeding disorders. All patients with ESA resistance on the hemodialysis service were included. To form the control group, patients who met the inclusion criteria were invited to participate. This group consisted of 40 patients with the same characteristics but without erythropoietin resistance, matched for age, in order to ascertain the possible association between erythropoietin resistance and telomere length.

### 4.2. Exclusion Criteria

Patients with moderate to severe smoking, active alcoholism at the time of the study, or uncontrolled systemic arterial hypertension. Patients with a history of autoimmune disease (lupus erythematosus, focal segmental glomerulosclerosis). All patients were invited to participate, informed consent was obtained, and a written informed consent form was signed.

The study complies with the Helsinki Declaration and was approved by the Institutional Ethical Committee of the Mexican Institute of Social Security (IMSS R-2017-501-027).

Telomere length was determined in peripheral blood leukocytes as a marker of cellular aging, in order to associate this parameter with erythropoietin resistance. In addition, other markers associated with erythropoietin resistance were quantified: hemoglobin levels, C-reactive protein, serum albumin, ferritin, serum iron, transferrin, parathyroid hormone, serum calcium, and phosphorus.

### 4.3. Technique for Telomere Length Measurement

DNA was extracted from circulating leukocytes, and telomere length was measured as previously described by PCR amplification using primers designed to hybridize with the TTAGGG and CCCTAA repeats. The final concentration of the PCR reagents was 0.2 Sybr Green I (Molecular Probes Roche), 15 mM Tris-HCl pH 8.0, 50 mM KCl, 2 mM MgCl_2_, 0.2 mM each dNTP, 5 mM DTT, 1% DMSO, and 1.25 U AmpliTaq Gold DNA polymerase. All PCRs were performed in a Light Cycler Real Time thermocycler. LightCycler^®^ (model 1.5) by Roche thermocycler. The cycles and temperature profile for both amplicons were as follows: 95 °C incubation for 3 min to activate the AmpliTaq Gold DNA polymerase. PCR for telomere: 40 cycles of 95 °C for 15 s, 54 °C for 2 min. For 36B4 PCR: 40 cycles of 95 °C for 15 s, 58 °C for 1 min. The technique used is based on that of Dr. Richard M. Cawthon [51], which employs 2 RT-PCRs: the first to amplify the telomeric segment (T) and the second to amplify the reference gene 36B4 (S), which encodes the acidic ribosomal phosphoprotein (located on chromosome 12). The T/S ratio is obtained to calculate telomere length.

### 4.4. Statistical Analysis

Statistical analysis was performed with SPSS 23.0 for Windows (SPSS Inc., Chicago, IL, USA). The Shapiro–Wilk test was used to assess the normal distribution of continuous variables. Descriptive statistics were performed for continuous variables. Differences between the group with resistance to erythropoiesis and the group without resistance to erythropoiesis for continuous and categorical characteristics were evaluated using Student′s *t*-test and chi-squared, respectively. A multivariate logistic regression analysis was performed, treating erythropoietin resistance as the dependent variable and including telomere length (T/S ratio) and other relevant clinical variables as covariates. Statistically different data were considered with *p*-values < 0.05.

## 5. Conclusions

Telomere shortening demonstrates a possible association with anemia and erythropoietin resistance in patients with CKD undergoing hemodialysis. This relationship suggests the need to explore whether telomere length recovery improves the response to ESAs.

## Figures and Tables

**Table 1:** General characteristics of patients with or without resistance to Erythropoiesis.

	Group with Resistance to Erythropoiesis n = 40	Group Without Resistance to Erythropoiesis n = 40	*p*-Value
Female gender %	25	22.5	*p* = 0.75
Age (years)	34 ± 14.7	38 ± 11.6	*p* = 0.87
Time on Hemodialysis (years)	5 ± 4.1	5 ± 3.3	*p* = 0.77
Erythropoeitin (U/week)	24,000 ± 4258	4000 ± 2783	*p* = 0.01

Data are presented as n = number (% = percentage), mean ± SD *p* < 0.05 considered significant.

**Table 2:** Laboratory values of patients with or without resistance to Erythropoiesis.

	Group with Resistance to Erythropoiesis n = 40	Group Without Resistance to Erythropoiesis n = 40	*p*-Value
T/S telomere length	0.45 ± 0.04	0.56 ± 0.03	*p* < 0.001
Hemoglobin (g/dL)	8.8 ± 2.1	11.6 ± 1.81	*p* < 0.001
Hematocrit (%)	28.9 ± 6.28	35.6 ± 5.62	*p* < 0.001
Iron (UG/DL)	115 ± 41.7	89.5 ± 58.1	*p* = 0.52
Iron saturation (%)	62.5 ± 26.6	46 ± 28.9	*p* = 0.76
Transferrin (mg/dL)	98 ± 33.2	97 ± 30.1	*p* = 0.85
Ferritin (ng/mL)	2059.4 ± 1289.5	2259 ± 1214	*p* = 0.64
Urea (mg/dL)	102.7 ± 32.6	98.4 ± 25.9	*p* = 0.75
Creatinine (mg/dL)	8.3 ± 3.4	10.1 ± 2.8	*p* = 0.21
Albumin (g/dL)	4.1 ± 0.71	4.4 ± 0.32	*p* = 0.04
Calcium (mg/dL)	8.7 ± 0.73	8.8± 0.92	*p* = 0.64
Phosphorus (mmol/dL)	5.05 ± 1.52	5.2 ± 1.49	*p* = 0.92
PTH pg/mL	788 ± 538.47	535.65 ± 603.06	*p* < 0.001
CRP mg/dL	3.6 ± 2.80	3.4 ± 2.34	*p* = 0.72

Data are presented as n= number (%= percentage), mean ± SD *p* < 0.05 considered significant. PTH: Parathyroid hormone, CRP: C-reactive protein.

## Data Availability

Data will be available upon request to the corresponding author.
